# A Series of Imidazole Derivatives: Synthesis, Two-Photon Absorption, and Application for Bioimaging

**DOI:** 10.1155/2015/965386

**Published:** 2015-10-12

**Authors:** Yingzhong Zhu, Lufei Xiao, Meng Zhao, Jiazheng Zhou, Qiong Zhang, Hui Wang, Shengli Li, Hongping Zhou, Jieying Wu, Yupeng Tian

**Affiliations:** ^1^Department of Chemistry, Key Laboratory of Functional Inorganic Material Chemistry of Anhui Province, Anhui University, Hefei 230601, China; ^2^Department of Food and Environmental Engineering, Chuzhou Vocational and Technical College, Chuzhou 239000, China; ^3^State Key Laboratory of Coordination Chemistry, Nanjing University, Nanjing 210093, China

## Abstract

A new series of D-*π*-A type imidazole derivatives have been synthesized and characterized. Two corresponding imidazolium salts (iodine and hexafluorophosphate) were prepared from the imidazole compound. Their electron-withdrawing ability can be largely tunable by salt formation reaction or ion exchange. UV-vis absorption and single-photon fluorescence spectra have been systematically investigated in different solvents. The two-photon cross sections (*δ*_2PA_) of the imidazole derivatives are measured by two-photon excited fluorescence (2PEF) method. Compared with those of **T-1** (107 GM) and **T-3** (96 GM), **T-2** (imidazolium iodine salt) has a large two-photon absorption (2PA) cross section value of 276 GM. Furthermore, the cytotoxicity and applications in bioimaging for the imidazole derivatives were carried out. The results showed that **T-1** can be used as a lysosomal tracker with high stability and water solubility within pHs of 4–6, while **T-2** and **T-3** can be used as probes for cell cytoplasm.

## 1. Introduction

Fluorescence biomarker has become a powerful tool for the monitoring and investigating of cellular processes in live tissues, as well as* in vivo* clinical related researches. Ideal fluorescence probes offered a unique approach for visualizing morphological details of tissue under subcellular resolution [1], without altering the biological activity of live cells/tissues in long-term range. For more specific cell biological research purpose, probes can be designed to bond with specific organelle of interest and provide guaranteed fluorescence signals. Until now, a variety of new fluorescence probes, such as organic dyes [2, 3], metal complexes [4, 5], and quantum dots [2, 3, 6, 7], have been synthesized for such purpose.

Compared to single-photon imaging, two-photon imaging shows significant benefits, such as deep tissue penetration and less photobleaching [8–11]. These superiorities encouraged researchers to make great efforts to obtain the materials with excellent two-photon absorption (2PA) properties in last decades. Generally, a large 2PA cross section is prerequisite, which is influenced by electron-donor and withdrawing abilities, conjugation length, and planarity of the *π* center [12–14]. Imidazole, as an N-heterocycle molecule, has been widely used in many biological processes [15–18]. It has high electron-withdrawing ability and good coplanarity, rendering it to be an ideal building block for nonlinear optical materials [19, 20]. In addition, triphenylamine group was utilized in optical material [21–23] due to a strong electron donor and effective conjugation length. Stryryl groups with excellent coplanarity are beneficial to increase effective conjugation length [24, 25]. Based on the above considerations, at present work, in order to obtain the material with large 2PA cross section, imidazole, triphenylamine, and styryl group were used as link to construct the three D-*π*-A chromophores. The photophysical properties and the connections between structure and properties of the three chromophores were investigated. Furthermore, potential biological applications of them were carried out.

## 2. Experiment

See Scheme 1.

### 2.1. General

All chemicals were commercially available and used without further purification. The solvents were purified by conventional methods before being used. 4-(1H-Imidazol-1-yl)benzaldehyde was synthesized according to the methods reported. The ^1^H-NMR and ^13^C-NMR spectra recorded at 25°C using Bruker Avance 400 spectrometer were reported as parts per million (ppm) from TMS. Mass spectra were determined with a Micromass GCT-MS (EI source).

X-ray diffraction data of single crystals were collected on CCD diffractometer. The determination of unit cell parameters and data collections were performed with Mo-K_*α*_ radiation (*λ* = 0.71073 Å). Unit cell dimensions were obtained with least-squares refinements, and all structures were solved by direct methods using SHELXS-97. The other nonhydrogen atoms were located in successive difference Fourier syntheses. The final refinement was performed by full-matrix least-squares methods with anisotropic thermal parameters for nonhydrogen atoms on F^2^. The hydrogen atoms were added theoretically and bonded with the concerned atoms.

Electronic absorption spectra were obtained on a UV-265 spectrophotometer. Fluorescence measurements were performed using a Hitachi F-7000 fluorescence spectrophotometer. For time-resolved fluorescence measurements, the fluorescence signals were collimated and focused onto the entrance slit of a monochromator with the output plane equipped with a photomultiplier tube (HORIBA HuoroMax-4P). The decays were analyzed by “least-squares.” The quality of the exponential fits was evaluated by the goodness of fit (*χ* ^2^). TPEF spectra were measured using femtosecond laser pulse and Ti: sapphire system (680–1080 nm, 80 MHz, 140 fs, Chameleon II) as the light source.

### 2.2. Synthesis

#### 2.2.1. 4-(1H-Imidazol-1-yl)benzaldehyde**  (**1**)**

4-(1H-Imidazol-1-yl)benzaldehyde was prepared according to the literature method [26].

#### 2.2.2. Synthesis of (E)-4-(4-(1H-Imidazol-1-yl)styryl)-N,N-diphenylaniline

*t*-BuOK (2.32 g, 20 mmol), 4-(1H-imidazole-1-yl)benzaldehyde** (1)** (1.72 g, 10 mmol), and 4-((iodotriphenyl phosphoranyl)methyl)-N,N-diphenylaniline (6.47 g, 10 mmol) 150 mL dry THF were added and mixed equably. The mixture was stirred for 10 min and then heated to 65°C for 24 h. The reaction was monitored by TLC. 250 mL CH_2_Cl_2_ was added after the solvent was removed. The organic layer was washed with water several times and dried over anhydrous MgSO_4_. The residue was purified by flash chromatography on silica gel using petroleum/ethyl acetate (5 : 1) as eluent and gave green solid** T-1** (1.7 g, yield: 47.2%). ^1^H-NMR: (DMSO, 400 MHz) *δ* (ppm) 8.30 (s, 1H), 7.78 (s, 1H), 7.70–7.72 (d, 2H, *J* = 8.4), 7.66–7.68 (d, 2H, *J* = 8.4), 7.52–7.54 (d, 2H, *J* = 8.4), 7.31–7.35 (t, 3H, *J* = 7.6), 7.25–7.31 (d, 1H, *J* = 16.4), 7.14–7.18 (d, 1H, *J* = 16.4), 7.19–7.21 (d, 2H, *J* = 9.6), 7.04–7.07 (t, 6H, *J* = 7.8), 6.96–6.98 (d, 2H, *J* = 8.4). ^13^C-NMR (DMSO, 400 MHz) *δ* (ppm): 146.90 (CH), 135.91 (CH), 135.37 (CH), 129.58 (CH), 127.65 (CH), 127.47 (CH), 125.63 (CH), 124.15 (CH), 123.29 (CH), 122.88 (CH), 120.36 (CH), 117.79 (CH). MS, *m*/*z*: 414.

#### 2.2.3. Synthesis of (E)-1-(4-(4-(Diphenylamino)styryl)phenyl)-3-methyl-1H-imidazol-3-ium Iodide**  **(**T-2**)

**T-1** (0.413 g, 1 mmol) and 5 mL CH_3_I were mixed together in 50 mL flask. The mixture was stirred overnight and filtered and it gave green solid (**T-2**) (0.48 g, yield: 85.6). ^1^H-NMR: (DMSO, 400 MHz) *δ* (ppm) 9.77 (s, 1H), 8.31 (s, 1H), 7.96 (s, 1H), 7.83–7.85 (d, 2H, *J* = 8), 7.75–7.77 (d, 2H, *J* = 8), 7.53–7.55 (d, 2H, *J* = 8), 7.32–7.36 (t, 5H, *J* = 7.6), 7.18–7.22 (d, 1H, *J* = 16), 7.05–7.11 (dd, 6H, *J* = 9.2,8), 6.97–6.99 (d, 2H, *J* = 8), 3.95 (s, 3H). ^13^C-NMR (DMSO, 400 MHz) *δ* (ppm): 147.18 (CH), 146.81 (CH), 138.81 (CH), 135.71 (CH), 133.18 (CH), 130.62 (CH), 130.03 (CH), 129.61 (CH), 128.29 (CH), 127.88 (CH), 127.56 (CH), 124.90 (CH), 124.31 (CH), 123.45 (CH), 122.61 (CH), 121.88 (CH), 120.75 (CH), 36.14 (CH_3_). MS, *m*/*z*: 429.

#### 2.2.4. Synthesis of (E)-1-(4-(4-(Diphenylamino)styryl)phenyl)-3-methyl-1H-imidazol-3-ium Hexafluorophosphate (V)**  **(**T-3**)

**T-2** (0.561 g, 1 mmol) and 100 mL CH_3_CN were added to flask with stirring for 10 min, followed by dropping AgPF_6_ (0.253, 1 mmol) which was dissolved in 30 mL CH_3_CN. The resulting suspension was further stirred for 2 hours before being filtrated. After removal of the solvent, the yellow-green solid was obtained (0.55 g, 96.0%). ^1^H-NMR: (DMSO, 400 MHz) *δ* (ppm) 9.77 (s, 1H), 8.31 (s, 1H), 7.96 (s, 1H), 7.83–7.85 (d, 2H, *J* = 8), 7.75–7.77 (d, 2H, *J* = 8), 7.53–7.55 (d, 2H, *J* = 8), 7.32–7.35 (t, 5H, *J* = 7.6), 7.18–7.22 (d, 1H, *J* = 16), 7.05–7.11 (dd, 6H, *J* = 9.2,8), 6.97–6.99 (d, 2H, *J* = 8), 3.95 (s, 3H). ^13^C-NMR (DMSO, 400 MHz) *δ* (ppm): 147.18 (CH), 146.81 (CH), 138.81 (CH), 135.70 (CH), 133.18 (CH), 130.61 (CH), 130.03 (CH), 129.61 (CH), 128.29 (CH), 127.87 (CH), 127.55 (CH), 124.88 (CH), 124.32 (CH), 123.45 (CH), 122.60 (CH), 121.88 (CH), 120.75 (CH), 36.11 (CH_3_). MS, *m*/*z*: 429.

## 3. Results and Discussion

### 3.1. Crystal Structures of**  T-1** ****and**** **T-3**

The single crystals of** T-1** and** T-2**, suitable for the X-ray analysis, were obtained from slow evaporation of methanol/benzene at room temperature several days later. Crystal data collection and refinement parameters are listed in Table S1 in Supplementary Material available online at http://dx.doi.org/10.1155/2015/965386. The crystal structures of** T-1** and** T-3** are shown in Figure S1.

**T-1** crystalizes with two independent molecules in asymmetric unit, and the crystal of** T-1** belongs to monoclinic system with* P2*_*1*_ */n* space group. The benzene rings of the triarylamine constitute a structure like propeller, with dihedral between two benzene rings 74.84°. The dihedral angle between styryl and benzene ring of triarylamine is 7.97°, while the dihedral angle between imidazole ring and styryl is 9.27°. What is more, all the bond lengths of C-C are located between the normal C=C double bond (1.32 Å) and C-C single bond (1.53 Å), which show a highly *π*-electron delocalized system in the molecule. With regard to** T-2**, it crystalizes in triclinic system with $P\overset{-}{1}$ space group and two independent molecules in asymmetric unit. Compared with** T-1,** the introduction of methyl leads to a larger dihedral angle; the dihedral angle between styryl and benzene ring of triarylamine is 18.80°, while between imidazole ring and styryl it is 22.79°. This means** T-1** has a better planarity of *π* center. The structural features indicate that the coplanarity of the two compounds is appropriate, which is a necessary condition for preferable nonlinear optical properties [27].

### 3.2. Linear Absorption and Single-Photon Excited Fluorescence

**Figure 1:**
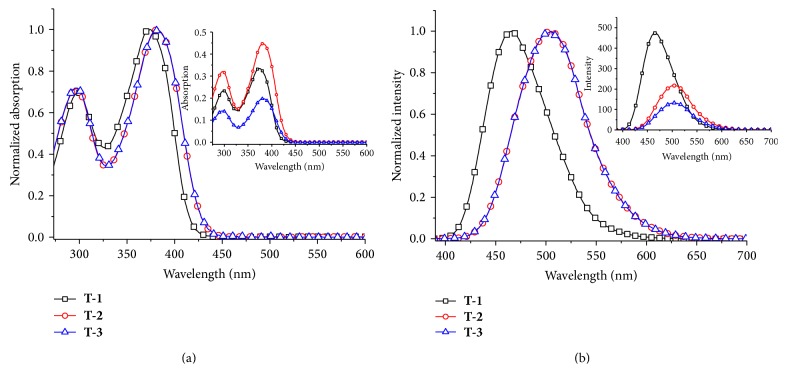
The normalized UV-vis spectra (a) and single-photon fluorescence spectra (b) of** T-1, T-2,** and** T-3 **(1.0 × 10^−5^ M) in DMF solution; the insert shows the original data of their UV-vis spectra and single-photon fluorescence spectra.

The UV-vis absorption spectra of the three chromophores** T-1, T-2,** and** T-3** in different solvents are shown in Figure S2. And the corresponding absorptive data are listed in Table 1. Their absorption spectra show peak maxima at ~295 and ~380 nm, with *ε* > 10^4^ dm^3^ mol^−1^ cm^−1^. There is only slight solvatochromic shift (±5 nm) for the absorption spectra of the three chromophores upon changing the solvents from benzene to DMF, indicating that the polarity of solvents has little effect on the ground state and the excited state [28].  Figure 1(a) depicts their UV-vis spectra in DMF. The high energy bands of all the chromophores occur at about 290 nm with little red-shift. The low energy bands appear around 380 nm. The normalized line of** T-2** coincides with that of** T-3**. This means the exchanging anion has no influence on the UV-vis absorption. Nevertheless, compared with** T-1**, imidazolium salts show small red-shift (10 nm) which may be caused by the formation of imidazolium cation as a stronger acceptor. Through calculation of molar absorption coefficient (log*ε*) the band at about 295 nm can be attributed to the *π* → *π* ^*^ transition from triphenylamine moiety, while the band around 480 nm is ascribed to *π* → *π* ^*^ transition within the entire molecule.

The single-photon fluorescence spectra of** T-1**,** T-2**, and** T-3** in different solvents are shown in Figure S3. The corresponding emission spectral data are listed in Table 1. It can be seen from Figure S3 that a remarkable bathochromic shift takes place for all the chromophores upon changing the solvent from benzene to DMF, which can be explained that the degree of charge separation in the excited state increase resulting in a larger dipole moment than that in ground state; therefore, the emission spectra of these dipolar chromophores exhibit sensitivity to solvent polarity [29, 30]. Accordingly, the Stokes shifts significantly increase with increasing solvent polarity, ranging from 3196 cm^−1^ in benzene to 5278 cm^−1^ in DMF (**T-1**), 3788 cm in benzene to 6513 cm^−1^ in DMF (**T-2**), and 3967 cm in benzene to 6444 cm^−1^ in DMF (**T-3**). The emission spectra of** T-1**,** T-2,** and** T-3** in benzene show a well resolved vibrational structure. When increasing solubility and solvent polarity, a loss of the vibronic structure is observed; a progressive red-shift of the emission wavelength occurs [27]. Compared to** T-1**, both imidazolium salts have an obvious red-shift over 20 nm in nonpolar solvent and 40 nm in polar solvent. This phenomenon was caused by the salt formation to enhance the electron accepted ability of the imidazole group.

The chromophores exhibit high quantum yield (>0.1) in various solvents. Importantly, the quantum yields (Φ) in high polar solvent DMF are 0.59 (**T1**), 0.23 (**T2**), and 0.45 (**T3**), respectively.** T1** with a weaker electron acceptor, imidazole ring, could cause the extra charge-separated in excited state [31], so** T-1** possesses the highest fluorescence quantum yield. The data of fluorescence lifetimes are listed in Table 1; it shows that the chromophores have similar lifetime in the same solvent. This can be attributed to nearly molecular stabilization effect on the excited state in the extended delocalization system of the molecules. With the increasing polarity of the solvents the lifetime prolongs from 1.7 to 2.54 ns for** T-1**, 1.66 to 2.14 ns for** T-2**, and 1.53 to 2.21 ns for** T-3**. That is to say, the conformational stability of the excited molecule is influenced by the polarity of the solvents [32].

**Figure 2:**
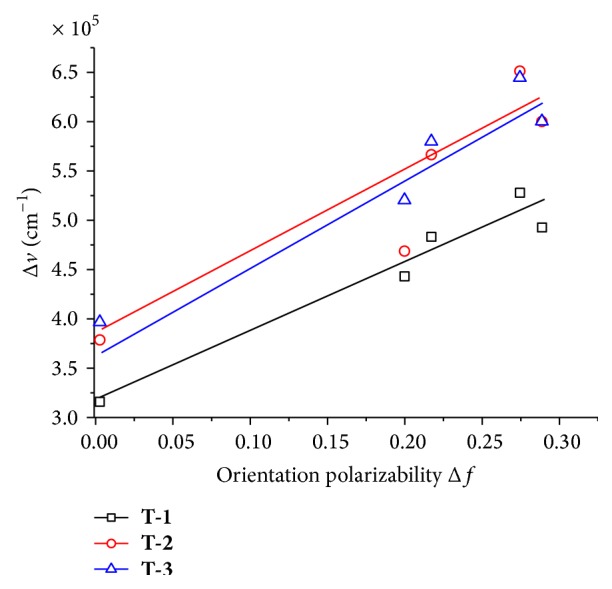
Lippert-Mataga plots for** T-1**,** T-2**, and** T-3**.

The Lippert-Mataga equation (shown in Supplementary Material) is widely used to evaluate the dipole moment changes of the dyes with photoexcitation [33, 34]; the emission of the chromophores, especially, is strongly dependent on solvent polarity. As shown in Figure 2, the Lippert-Mataga plots exhibit a linear behavior; this means no specific interaction exists between the solvent and the chromophores, except for the polarizability as modeled [35, 36]. The slop of the fitting line is related to the dipole moment change between the ground and excited states (*μ*_e_ − *μ*_g_). Larger slope in the Lippert-Mataga plot infers larger dipole moment changes with photoexcitation. The dipole moment changes of the chromophores** T-1**,** T-2,** and** T-3**  (*μ*_e_ − *μ*_g_) are calculated as 13.07 D, 13.70 D, and 14.47 D, respectively [37]. This means the chromophores in the excited state have large polar structures which provide promising linear and nonlinear optical properties [38].

### 3.3. Two-Photon Excited Fluorescence (2PEF)

2PA cross sections of the compounds were determined by two-photon excited fluorescence (2PEF) method in near-IR (NIR) range from 700 to 1000 nm. Experiments revealed that linear absorption did not exist from 500 nm to 900 nm for all the chromophores, indicating that there were no molecular energy levels corresponding to an electron transition in this spectral range. Hence, upon excitation from 700 nm to 1000 nm at intervals of 10 nm (similarly hereinafter), it was impossible to produce single-photon excited upconverted fluorescence. If frequency upconverted fluorescence appeared upon excitation with a tunable laser in this range, it should be safely attributed to multiphoton excited fluorescence.

**Figure 3:**
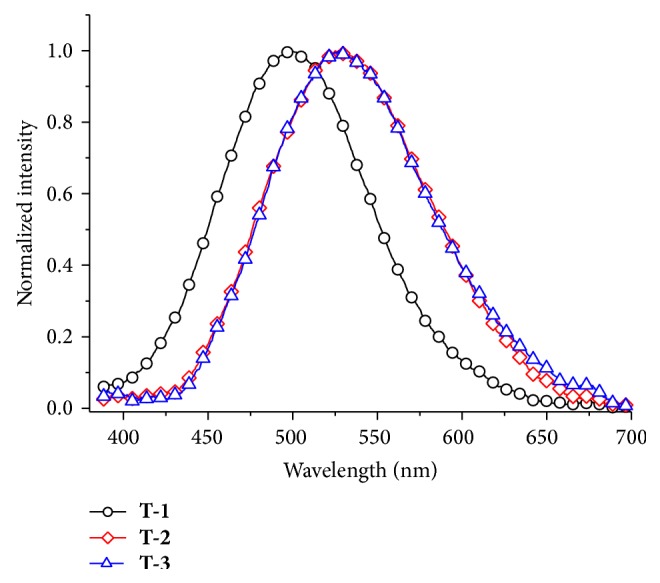
The normalized two-photon excited fluorescence spectra of** T-1**,** T-2,** and** T-3** (excitation wavelength at 760 nm, energy of 500 mW, *c* = 1.0 × 10^−3^ mol·L^−1^) in DMF.

By tuning the pump wavelengths incrementally from 700 to 880 nm, keeping the input power fixed, the 2PEF intensities were recorded. As shown in Figure S5 all of the chromophores exhibit fluorescence in the wavelength range of 720–880 nm. Figure S4 shows the linear dependence on the square of input laser power which suggests a two-photon excitation mechanism at 760 nm for the chromophores. The normalized 2PEF peak positions of** 3P-2** and** 3P-3** shown in Figure 3 show no difference, which means anions have little influence on the position. But anions can influent their intensity of 2PEF which shown in Figure S6. Nevertheless compared with** T-1**, imidazolium salts show a clear red-shift for near 30 nm.

**Figure 4:**
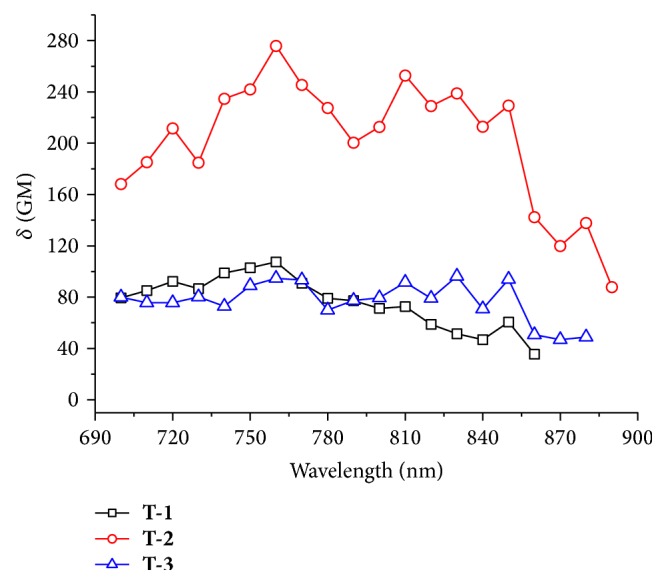
Two-photon absorption cross sections of** T-1**,** T-2,** and** T-3** (excitation wavelength from 700 nm to 890 nm, power of 500 mW, *c* = 1.0 × 10^−3^ mol·L^−1^) in DMF.

The two-photon absorption (2PA) cross section (*σ*) of the chromophores** T-1**,** T-2,** and** T-3** in DMF using two-photon-induced fluorescence method with fluorescein as the standard is shown in Figure 4 [39, 40]. The 2PA cross section equation is shown in Supplementary Material. The largest 2PA cross sections of all the chromophores located at 760 nm with the highest values 107 GM, 276 GM, and 96 GM, respectively, are depicted in Figure 4.** T-2** owns the largest 2PA cross section in this spectral range, though** T-1** has the largest two-photon fluorescence intensity in Figure 3. The reason is that maybe** T-2** has the smallest fluorescence quantum yield.

### 3.4. Cell Image and Cytotoxicity Assay

Due to their high quantum yield, long fluorescence lifetime, and large 2PA cross section, these three chromophores potentially can be utilized in bioimaging.

**Figure 5:**
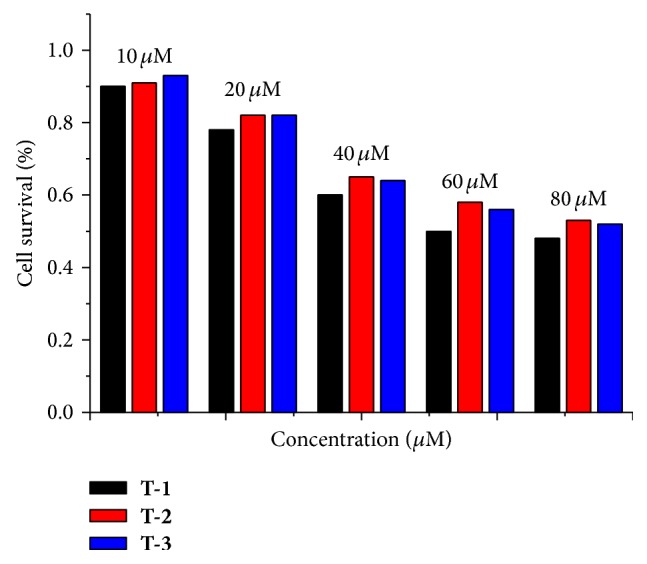
Cytotoxicity data results of** T-1**,** T-2**, and** T-3** obtained from the MTT assay.

Before exploring their biological applications, cytotoxicities of the chromophores were firstly measured toward the human cervical carcinoma cells (HepG2) by MTT assay, a standard method to probe cell survival fraction.  Figure 5 shows that, in the presence of the probes with the concentration from 10 to 20 *μ*M, the cellular viabilities of HepG2 cells are greater than 85% after incubation for 24 h. All of the data indicate that low-micromolar concentrations of three probes are essentially low-nontoxic within 24 h. As a result, all the chromophores can safely be used for further bioimaging.

**Figure 6:**
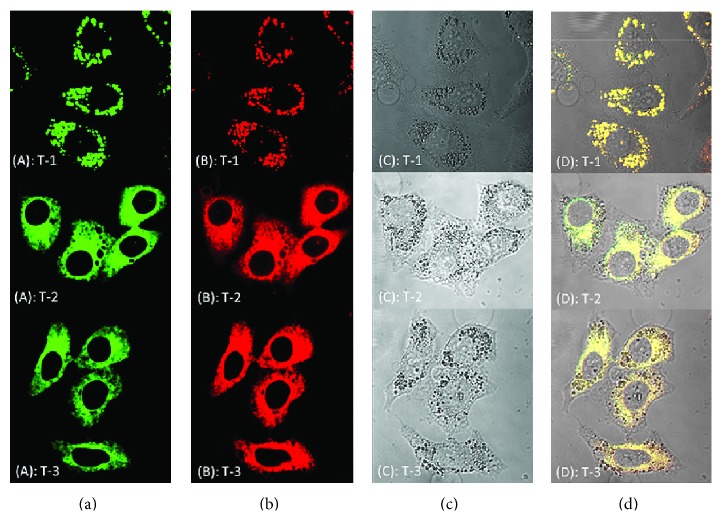
(a) One-photon image of HepG2 cells incubated with 20 *μ*M after 20 min of incubation, washed by PBS buffer. (b) Two-photon image of HePG2 cells incubated with 20 *μ*M after 30 min of incubation, washed by PBS buffer. (c) Bright field image of HePG2 cells. (d) The overlay of (a) to (c). Scale bars represent 10 *μ*M.

Afterwards, fluorescent images of confocal microscopy and two-photon microscopy of HepG2 cells labeled with the three fluorescent probes were captured, along with differential interference contrast (DIC) micrographs. As shown in Figure 6, HepG2 cells have successfully uptaken three fluorescent probes and clearly emerged from cellular cytoplasm suggesting that the complexes penetrate the phospholipids bilayer of cellular membrane and closely associated with some parts of the cell.

Confocal fluorescence imaging reveals that** T-1** exhibited observable punctate fluorescence around the perinuclear regions in the cell. To further confirm the** T-1** subcellular distribution, a commercially available lysosome-specific staining probe (Figure S8) was used to costain cells with** T-1**. It showed that the distribution of** T-1** and Lysotracker were highly overlapped (overlapping degree 0.918, measured via ImageJ Plugin, Colocalization Finder), suggesting that** T-1** might have higher affinity binding/aggregate within cellular lysosome. Lysosomes were known to have proton-pumping vacuolar ATPases, which maintain the internal microenvironment at a pH range of 4.6–6.0. As shown in Figure S7, due to the low solubility in water, the fluorescence intensity of** T-1** is only about 50 in aqueous solution with pH = 7. However, when pH in the solution decreases to a range of 3.0–6.0, the fluorescence intensity increases to near 200 and stays with slight change. This means that** T-1** has relatively higher stability and solubility in this pH range. On this basis we speculated that** T-1** selectively concentrated in lysosomes might cause by the protonation of exposed nitrogen atom of imidazole ring, which has parallel mechanism to commercially available lysosome-specific staining probe [41].

For the imidazolium salts (**T-2** and** T-3**) uptake, it showed different cellular destination. The intense fluorescence was mainly evenly distributed in the HepG2 cell cytoplasm and excluded from nuclear region. This suggested the chromophores could label the cell cytoplasm of HepG2, due to their more hydrophobic nature, therefore the chromophores behaved higher distribution degree within aqueous intracellular microenvironment. This binding property might provide a useful tool for monitoring and investigating intracellular process, such as cargo sorting, organelle movement, cell division, and vesicular transportation in live tissue.

## 4. Conclusion

In this contribution, three imidazole derivatives were designed and synthesized. UV-vis absorption, single-photon fluorescence, two-photon absorption characters, and two-photon fluorescence microscopy (2PFM) are systematically investigated. It was discovered that there is almost no influence on the UV-vis absorption of the chromophores while varying with the polarity of the solvents. However, an obvious influence on their fluorescent spectra can be observed. On account of large 2PA cross sections in the near-IR region, the imidazolium derivatives can selectively stain cell well which make them potential bioimaging applications in the future.

## Supplementary Material

Supplementary material shows the crystal data and crystal structures, the UV-vis absorption and single-photon fluorescence in different solution, Fluorescence quantum yield equation, Lippert-Mataga equation, Two photon absorption cross section equation, output fluorescence (I_0_) versus the square of input laser power (I_F_)^2^ at 760 nm for three chromophores in DMF, Two-photon excited fluorescence (TPEF) in DMF in all wavelength, TPEF in DMF at wavelength of 760 nm, the stability in different pH solution, and the co-localizes to lysosomes in cells.

## Figures and Tables

**Scheme 1:**
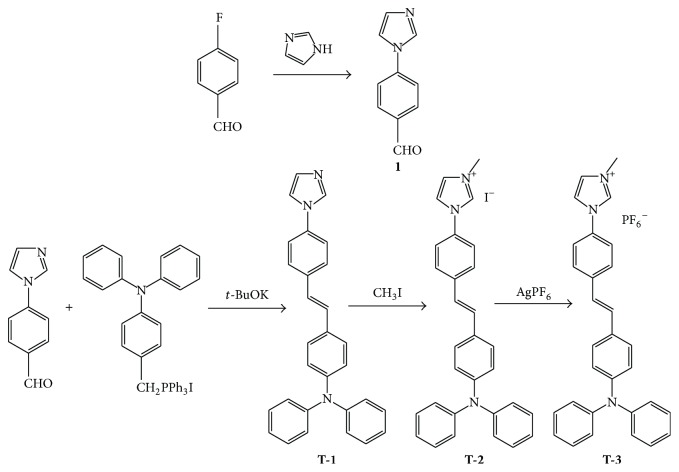
Synthesis routes for compounds** T-1**,** T-2**, and** T-3**.

**Table 1:** Photophysical properties of **T-1**, **T-2,** and **T-3** in several of different polar solvents.

Compound	Solvent	***λ***_**ab**_ (nm)^[a]^	log⁡*ε*	***λ***_**em**_ (nm)^[b]^	Φ^[c]^	**τ** (ns)^[d]^	Stokes' shift (cm^−1^)^[e]^
**T-1**	Benzene	299, 377	4.39, 4.55	428	0.61	1.71	10080, 3160
	DCM	296, 375	4.37, 4.52	458	0.67	2.28	11949, 4832
	Ethanol	294, 371	4.42, 4.56	454	0.59	2.33	11987, 4927
	Ethyl acetate	296, 371	4.43, 4.54	444	0.37	1.97	11261, 4431
	DMF	297, 374	4.37, 4.53	466	0.59	2.54	12210, 5278

**T-2**	Benzene	297, 383	4.46, 4.46	448	0.37	1.66	11348, 3788
	DCM	296, 389	4.47, 4.55	499	0.28	1.86	13743, 5666
	Ethanol	294, 381	4.50, 4.64	494	0.18	1.37	13770, 6003
	Ethyl acetate	294, 375	4.50, 4.56	455	0.35	1.84	12035, 4688
	DMF	296, 380	4.51, 4.65	505	0.23	2.14	13981, 6513

**T-3**	Benzene	298, 389	4.15, 4.19	460	0.69	1.53	11817, 3967
	DCM	295, 393	4.18, 4.27	509	0.42	1.97	14251, 5798
	Ethanol	294, 381	4.27, 4.40	494	0.33	1.37	13770, 6003
	Ethyl acetate	294, 377	4.36, 4.45	469	0.47	1.92	12691, 5203
	DMF	296, 381	4.16, 4.30	505	0.45	2.21	13981, 6444

^[a]^Peak position of the longest absorption band. ^[b]^Peak position of SPEF, excited at the absorption maximum. ^[c]^Quantum yields determined by using quinine sulfate as standard. ^[d]^Fluorescent lifetime in different solutions. ^[e]^Stokes' shift in cm^−1^.
