# Supplementary material for: A Series of Imidazole Derivatives: Synthesis, Two-Photon Absorption, and Application for Bioimaging
Source: Biomed Res Int. 2015 Oct 12;2015:965386. doi: 10.1155/2015/965386 (PMC4633690; doi:10.1155/2015/965386)
Supplement: Supplementary file 1 — Supplementary material shows the crystal data and crystal structures, the UV-vis absorption and single-photon fluorescence in different solution, Fluorescence quantum yield equation, Lippert-Mataga equation, Two photon absorption cross section equation, output fluorescence (I0) versus the square of input laser power (IF)2 at 760 nm for three chromophores in DMF, Two-photon excited fluorescence (TPEF) in DMF in all wavelength, TPEF in DMF at wavelength of 760 nm, the stability in different pH solution, and the co-localizes to lysosomes in cells. [file 965386.f1.pdf]

# Supporting Information

Table S1. The crystal data collection and the refinement parameters of T-1 and T-3 .. 2

Figure S1. The crystal structures of T-1(left) and T-3(right). H atoms, anions and solvents molecules have been omitted for clarity ...................................................... 4

Figure S2. UV-vis spectra of T-1, T-2 and T-3 ($1\times10^{-5}$ M) in different solvents......... 4

Figure S3. Single-photon fluorescence spectra of T-1, T-2 and T-3 ($1.0\times10^{-5}$M) in different solvents ........................................................................................ 5

Fluorescence quantum yield ........................................................................ 5

The Lippert-Mataga equation ...................................................................... 5

Two photon absorption cross section ($\delta$)................................................................. 6

Figure S4. Output fluorescence ($I_0$) vs. the square of input laser power $(I_F)^2$ at 760nm for T-1, T-2 and T-3 in DMF ........................................................................ 7

Figure S5. Two-photon exited fluorescence (TPEF) of T-1, T-2 and T-3 in DMF ...... 6

Figure S6. the two-photon excited fluorescence spectra of T-1, T-2 and T-3 (excitation wavelength at 760 nm, energy of 500 mW, c = $1.0\times10^{-3}$ molL-1) in DMF 8

Figure S7. Single-photon fluorescence spectra of T-1 ($1.0\times10^{-5}$M) with different pHs in water ............................................................................................................ 8

Figure S8. T-1 co-localizes to lysosomes in live HepG2 cells .................................... 7

Reference ........................................................................................................ 8

**Table S1.** The crystal data collection and the refinement parameters of T-1 and T-3

| | T-1 | T-3 |
|---|---|---|
| Empirical formula | C H1.73 N O0.03 | C36 H32 F6 N3 P |
| Formula weight | 28.2 | 651.62 |
| Temperature | 296(2)K | 298(2)K |
| Wavelength | 0.71073Å | 0.71069 Å |
| Crystal system, space group | Monoclinic, $P2_1/n$ | Triclinic, $P\bar{1}$ |
| *a*/Å | 9.61(13) | 10.37(5) |
| *b*/Å | 9.26(13) | 14.01(5) |
| *c*/Å | 55.98(8) | 24.32(5) |
| Volume | 4975(12) $Å^3$ | 3257(2) $Å^3$ |
| Z, Calculated density | 146, 1.374 $Mg/m^3$ | 4, 1.329 $Mg/m^3$ |
| Absorption coefficient | 0.097 $mm^{-1}$ | 0.149 $mm^{-1}$ |

| | | |
|---|---|---|
| F(000) | 2182 | 1352 |
| Crystal size | 0.30 ×0.20 × 0.10 mm | 0.30 ×0.20 × 0.10 mm |
| Theta range for date collection | 1.46 to 25.00 deg. | 1.66 to 25.00 deg. |
| Limiting indices | -11<=h<=11, -10<=k<=10, -66<=l<=66 | -12<=h<=11, 16<=k<=16, -28<=l<=27 |
| Reflections collected/unique | 29779 / 8315 [R(int) = 0.0780] | 23007 / 11327 [R(int) = 0.0253] |
| Completeness to theta=25.00 | 95.1 % | 98.6 % |
| Absorption correction | Semi-empirical from equivalents | Semi-empirical from equivalents |
| Max. and min. transmission | | 0.9853 and 0.9567 |
| Refinement method | Full-matrix least-squares on $F^2$ | Full-matrix least-squares on $F^2$ |
| Data / restraints / parameters | 8315 / 155 / 655 | 11327 / 0 / 940 |
| Final R indices [I>2sigma(I)] | R1 = 0.1147, wR2 = 0.3099 | R1 = 0.0953, wR2 = 0.2858 |
| R indices (all data) | R1 = 0.1814, wR2 = 0.3488 | R1 = 0.1442, wR2 = 0.3257 |

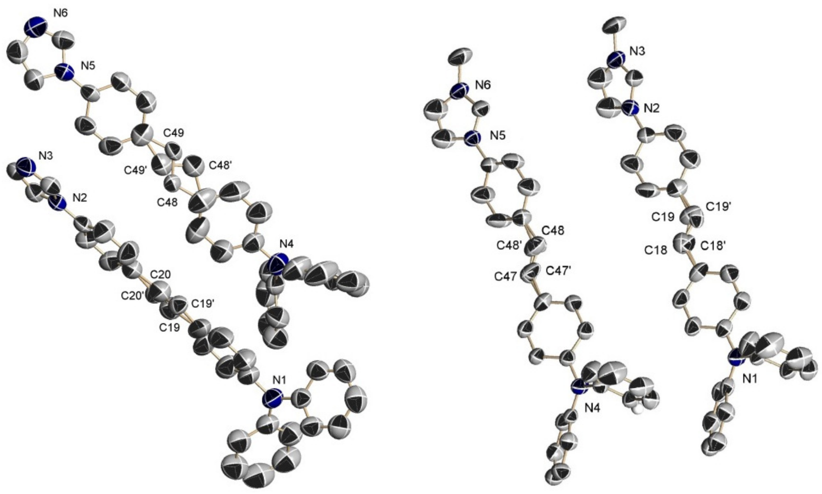


**Figure S1.** The crystal structures of T-1(left) and T-3(right). H atoms, anions and solvents molecules have been omitted for clarity

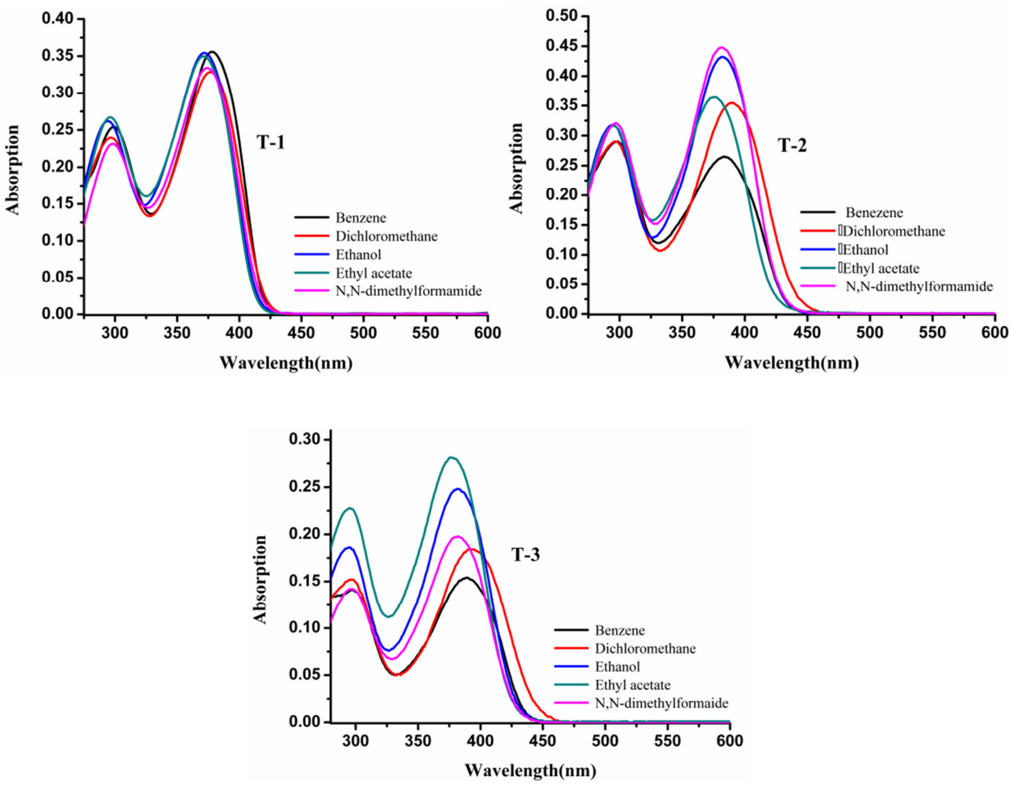


**Figure S2.** UV-vis spectra of **T-1, T-2** and **T-3** ($1\times10^{-5}$ M) in different solvents

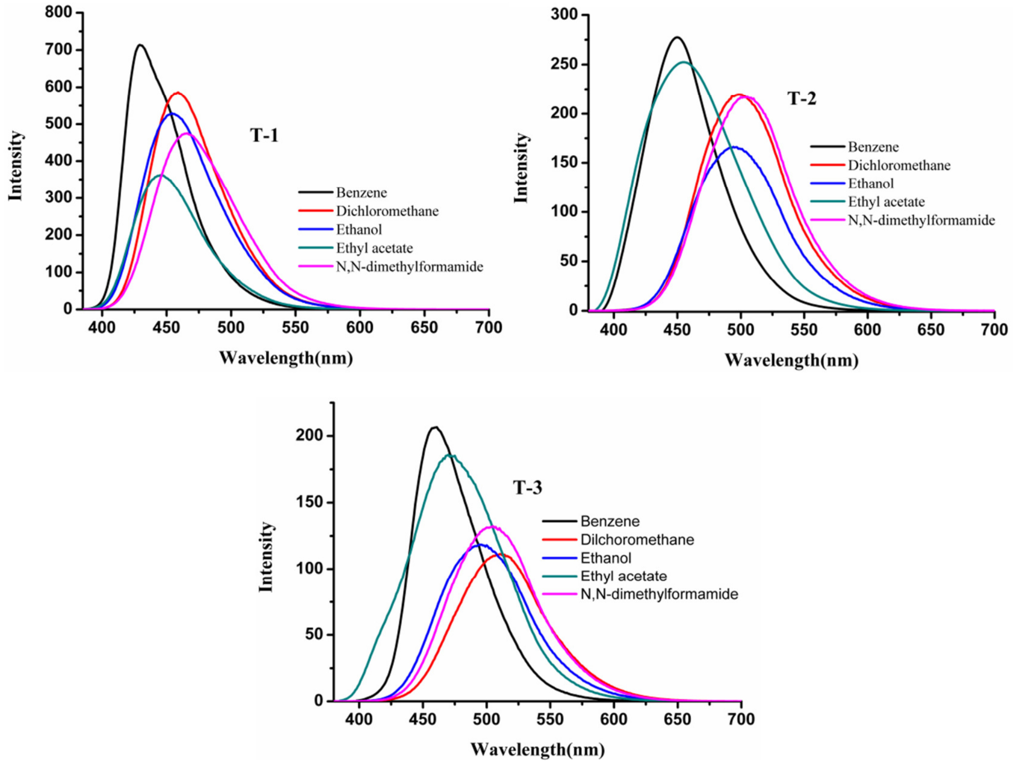


**Figure S3.** Single-photon fluorescence spectra of **T-1, T-2** and **T-3** ($1.0\times10^{-5}$M) in different solvents

## Fluorescence quantum yield

The fluorescence quantum yields (*Φ*) were determined by using Rhodamine-B as the reference according to the literature method [1]. Quantum yields is corrected as follows:

$$\Phi_s = \Phi_r \frac{A_r \eta_s^2 D_s}{A_s \eta_r^2 D_r}$$

where the *s* and *r* indices designate the sample and reference samples, respectively, *A* is the absorbance at $\lambda_{exc}$, $\eta$ is the average refractive index of the appropriate solution, and *D* is the integrated area under the corrected emission spectrum[2] .

## The Lippert-Mataga equation

The Lippert-Mataga equation is as follow:

$$\Delta \nu = \frac{2\Delta f}{4\pi\varepsilon_0 \hbar c a^3}(\mu_e - \mu_g)^2 + b$$

$$\Delta f = \frac{\varepsilon - 1}{2\varepsilon + 1} - \frac{n^2 - 1}{2n^2 + 1}$$

in which $\Delta v = v_{abs} - v_{em}$ stands for Stokes shift, $v_{abs}$ and $v_{em}$ are absorption and emission frequency ($cm^{-1}$), $\hbar$ is the Planc's constant, *c* is the velocity of light in vacuum, *a* is the Onsager radius and *b* is a constant. $\Delta f$ is the orientation polarizability, $\varepsilon$ is the refractive index, *n* is the dielectric constant, $\mu_e$ and $\mu_g$ are the dipole moments of the emissive and ground states, respectively and $\varepsilon_0$ is the permittivity of the vacuum. $(\mu_e\text{-}\mu_g)^2$ is proportional to the slope of the Lippert-Mataga plot.

### Two photon absorption cross section ($\delta$)

2PA cross sections ($\delta$) of samples is determined by Eq.:

$$\delta = \delta_{ref} \frac{\Phi_{ref}}{\Phi} \frac{c_{ref}}{c} \frac{n_{ref}}{n} \frac{F}{F_{ref}}$$

Where the '*ref*' subscript represents the reference molecule (here fluorescein in aqueous solution of sodium hydroxide (pH = 11) at a concentration of $1.0 \times 10^{-3}$ mol $L^{-1}$ was used as the reference). $\delta$ is the TPA cross-sectional value, *c* is the concentration of the solution, *n* is the refractive index of the solution, *F* is the 2PEF integral intensities of the solution emitted at the exciting wavelength, and $\Phi$ is the fluorescence quantum yield. The $\delta_{ref}$ value of reference was taken from the literature[3].

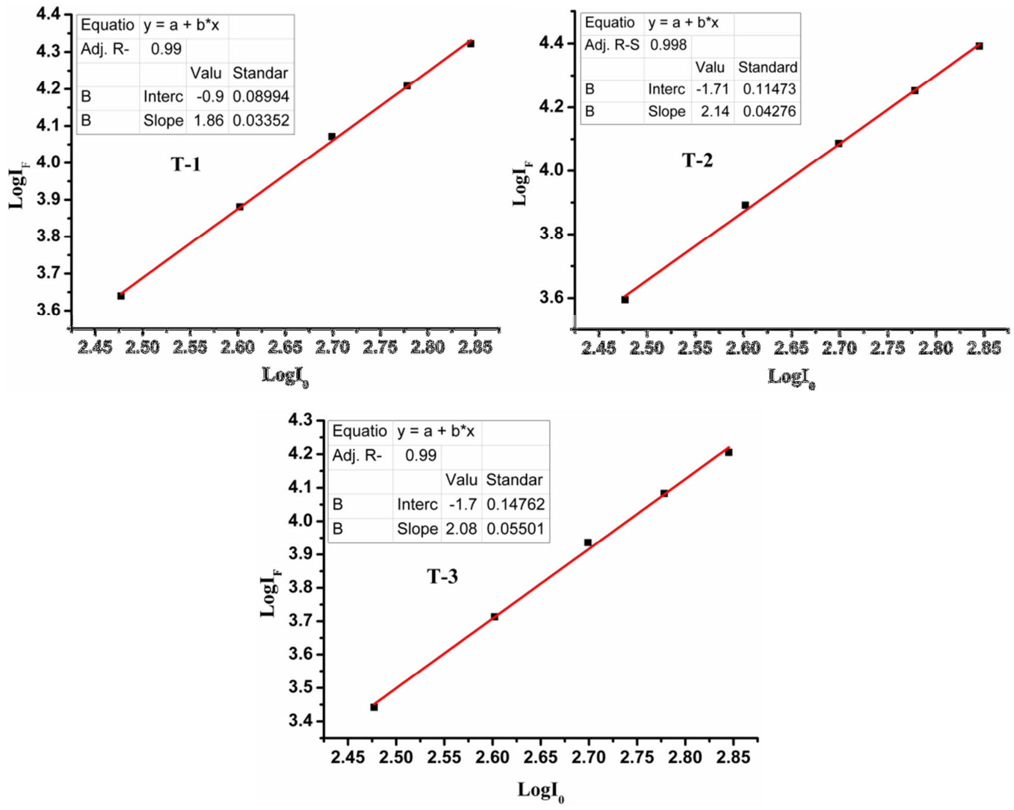


**Figure S4.** Output fluorescence ($I_0$) vs. the square of input laser power $(I_F)^2$ at 760nm for **T-1, T-2** and **T-3** in DMF

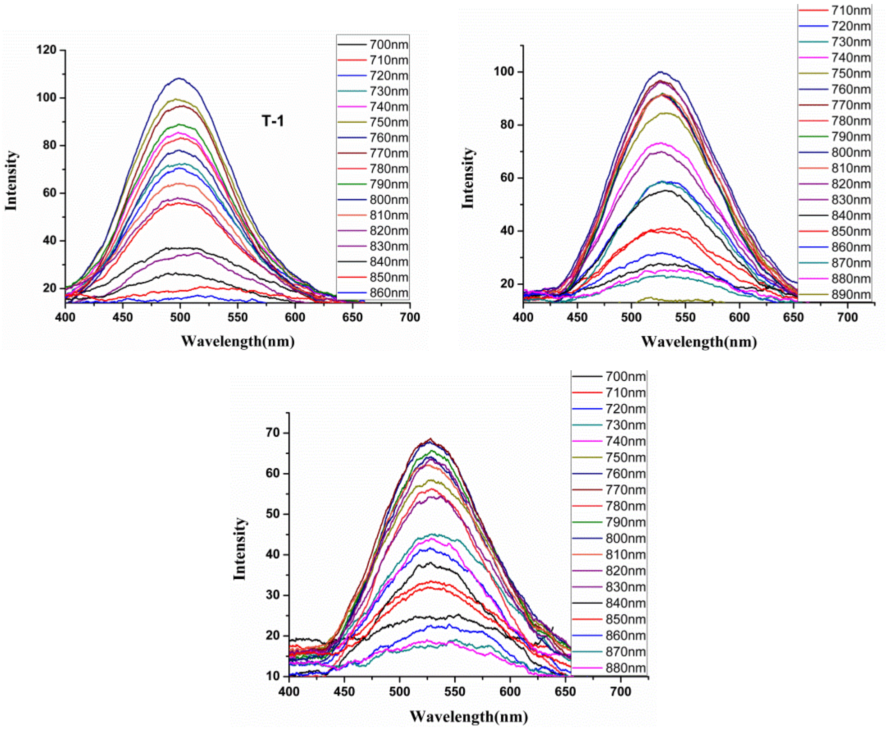

**Figure S5.** Two-photon exited fluorescence (TPEF) of **T-1**, **T-2**, and **T-3** in DMF

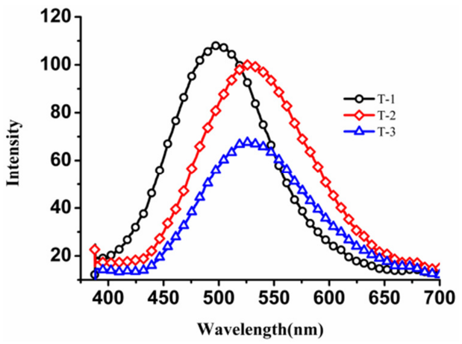


**Figure S6.** the two-photon excited fluorescence spectra of **T-1**, **T-2** and **T-3** (excitation wavelength at 760 nm, energy of 500 mW, c = 1.0×10$^{-3}$ molL-1) in DMF

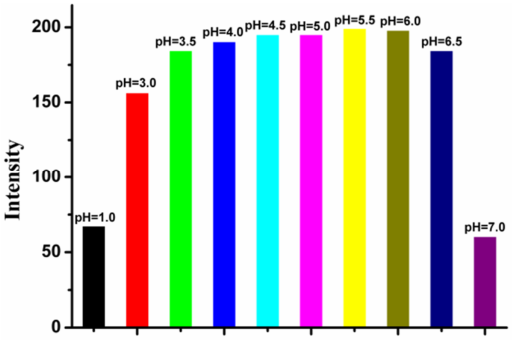


**Figure S7**. Single-photon fluorescence spectra of **T-1** (1.0×10$^{-5}$M) with different pHs in water

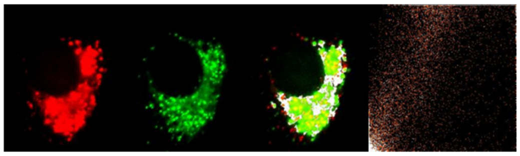

**Figure S8. T-1** co-localizes to lysosomes in live HepG2 cells

## Reference


[1] a. Kubin RF, Fletcher AN. Fluorescence quantum yields of some rhodamine dyes[J]. Journal of Luminescence, 1983; 27(4): 455-462. b. Karstens T, Kobs K. Rhodamine B and

rhodamine 101 as reference substances for fluorescence quantum yield measurements. The Journal of Physical Chemistry, 1980; 84(14): 1871-1872.

[2] Gray TG, Rudzinski CM, Meyer EE, Holm R, Nocera DG. Spectroscopic and photophysical properties of hexanuclear rhenium (III) chalcogenide clusters. Journal of the American Chemical Society. 2003;125(16):4755-70.

[3] He GS, Tan LS, Zheng Q, Prasad, P. N. Multiphoton absorbing materials: molecular designs, characterizations, and applications. *Chemical reviews*, 2008; *108*(4): 1245-1330.
